# Agomelatine, A Potential Multi-Target Treatment Alternative for Insomnia, Depression, and Osteoporosis in Postmenopausal Women: A Hypothetical Model

**DOI:** 10.3389/fpsyt.2021.654616

**Published:** 2021-06-29

**Authors:** Ahmet Yardimci, Mehmet Ridvan Ozdede, Haluk Kelestimur

**Affiliations:** Department of Physiology, Faculty of Medicine, Firat University, Elazig, Turkey

**Keywords:** menopause, insomnia, depression, osteoporosis, melatonin, agomelatine

## Abstract

Insomnia, which is associated with menopausal depression, is a common symptom of menopause. Both symptoms have a common etiology, and can affect each other significantly. Pharmacological interventions, including hypnotics and antidepressants, and non-pharmacological therapies are generally administered in clinical practice for insomnia treatment. As another menopausal disorder, osteoporosis is described as a disease of low bone mineral density (BMD), affecting nearly 200 million women worldwide. Postmenopausal osteoporosis is common among middle-aged women. Since postmenopausal osteoporosis mainly results from low estrogen levels, menopausal hormone therapy (HT) is considered the first-line option for the prevention of osteoporosis during the menopausal period. However, almost no study has evaluated novel treatments for the combined prevention of insomnia, depression, and osteoporosis. Hence, it is necessary to develop new multi-target strategies for the treatment of these disorders to improve the quality of life during this vulnerable period. Melatonin is the major regulator of sleep, and it has been suggested to be safe and effective for bone loss therapy by MT-2 receptor activity. As a result, we hypothesize that agomelatine, an MT-1 and MT-2 receptor agonist and 5-HT_2C_ receptor antagonist, holds promise in the combined treatment of insomnia, depression, and osteoporosis in middle-aged women during menopause.

## Introduction

Menopause, described as the termination of menstruation, indicates a significant transition regarding reproductive status in women. The menopausal transition (MET), starting 4–6 years before the discontinuation of menses (1), is associated with hormonal fluctuations and physiological and psychological symptoms, such as sleep disturbances, hot flashes (HF), and mood changes—the frequency, severity, and duration of which may vary among women (2). Among these symptoms, as a climacteric vasomotor symptom (VMS) (3), HF is a criterion for MET and has been reported to occur in about 80% of women (1). Hot flashes includes a sensation of heat, sweating, and chills (4). Globally, about 470 million women are in the postmenopausal phase; the number of which increases by 1.5 million each year, and it is expected to reach 1.2 billion by 2030. Approximately 50–85% of these women will experience VMS associated with menopause (5). In several studies, the relationship between sleep disorders and VMS has been investigated. Findings of a previous study, conducted among 962 midlife women, revealed a marked relationship between sleep disorders and VMS. About 81.3% of these women had severe VMS and poor-quality sleep; and 43.8% of them met the criteria for chronic insomnia (6). Additionally, the severity of insomnia was reported to have a correlation with HF and night sweats, and these symptoms are associated with a large-scale possibility of a diagnosis of insomnia (28.5% in women with HF and 10.5% in women without HF) (6). In accordance with the “domino effect theory,” HF disrupts sleep and leads to insomnia, which then increases vulnerability to depression. As a result, insomnia follows sleep disruption, and depression follows insomnia in a vicious cycle (6, 7). Additionally, Caruso et al. (2) reported that age-related irregularities in circadian rhythm may have a role as a mechanism preceding both sleep and mood disorders.

## Bidirectional Relationship Between Insomnia and Major Depression in Menopause

Clinical depression (including major depression) is known to be widespread with lifelong rates for women being 1.5–2 times higher than those for men. It is a complicated disorder, having several factors relevant to emotional, physical, and functional morbidity (8). Women may be at increased risk of depression during hormonal periods, such as MET, puberty, and pregnancy (9); and depressive symptoms are known to worsen throughout MET (1). Insomnia is described as a difficulty in initiating or sustaining sleep or the feeling that sleep is non-restorative, despite adequate opportunity for sleep (10). Insomnia is suggested to have a close relationship with depression and depressive disorders along the MET period (4). Insomnia symptoms are one of the most common complaints reported during the peri- and post-menopausal periods (11). Approximately 25–30% of the population have symptoms of insomnia, and this rate increases to 39–60% among women in the peri-menopausal period. Insomnia has been shown to increase the risk of developing depressive symptoms by 2–3 times during the peri-menopausal period of a woman's life (2), and more importantly, it often triggers depression (11). A 13-year mental health longitudinal study by the Study of Women's Health Across the Nation (SWAN) revealed that insomnia may have a contribution to the permanence and relapse of major depressive disorder (MDD) throughout menopause (8). Hence, it seems that women having sleep problems at MET may be at risk of depression in subsequent years. Disturbed sleep, a major criterion of depression according to the Diagnostic and Statistical Manual of Mental Disorders, Fifth Edition (DSM-5), is prevalent among women having clinical depression (8). Considering this relationship between insomnia and depression, women with menopausal insomnia seem likely to be at greater risk of depression due to the additional burden of poor sleep. Thus, the determination of safe and effective treatments for menopause-related insomnia disorders that also ameliorate comorbid depressive symptoms is advisable (11). All these results indicate that alleviating insomnia would improve not only insomnia symptoms but also depressive symptoms. Tal et al. (5) reported that treatment for sleep disturbance enhanced treatment of depression or alleviated depressive symptoms altogether.

## Insomnia Treatment by Non-hormonal Pharmacological Options

For the diagnosis and treatment of insomnia, cognitive behavioral therapy for insomnia (CBT-I) is proposed as the first-line treatment for chronic insomnia in adults of any age in the general population. However, pharmacological alternatives are recommended when CBT-I is not effective or available (7). These options include benzodiazepines, non-benzodiazepine hypnotics (Z-drugs), antidepressants, antipsychotics, and melatonin as non-hormonal pharmacological options (12). Additionally, menopausal hormone therapy (HT) is effective for sleep disorders in menopausal women (7). However, results based on several clinical studies investigating the effects of HT on insomnia in menopause are inconsistent (13), and numerous factors are involved in this treatment process, including the range of treatment protocols, dosages, and formulations (14). More importantly, a recent meta-analysis involving a search of 424 defined articles, from which 42 trials, showed that only menopausal women with VMS have an improvement in the quality of life with HT (15). Therefore, non-hormonal interventions, including antidepressants, seem likely to be better for use in this context for treating insomnia in this patient population. The use of antidepressants is not recommended in the treatment of menopause-related insomnia unless depression is present (12). Given that insomnia and depression have high comorbidity (16, 17) and shared etiology (18, 19), gold standard treatments for insomnia relevant to menopause should also ideally treat comorbid depressive symptoms in peri- and post-menopausal women at risk for depression (11). As mentioned before, since insomnia is the main criterion for depression according to DSM-5, insomnia treatment in menopausal women is also of great importance for the treatment of depression. Additionally, it would provide an alternative strategy for the indirect treatment of osteoporosis, which has a direct relationship with depression during menopause.

## The Effect of Depression on Menopause-Induced Osteoporosis

Osteoporosis is defined as a disease of low bone mineral density (BMD) or excessive bone loss, affecting 200 million women worldwide (20). Postmenopausal osteoporosis is prevalent in middle-aged women. Half the population of postmenopausal women have been suggested to suffer from an osteoporotic fracture in their lifetime, and those who have had a fracture are at high risk of subsequent fractures (21). More importantly, fractures resulting from osteoporosis are known to be more widespread than stroke, myocardial infarction, and breast cancer, combined. For women aged 50 years, the risk of a fracture due to osteoporosis is 50% in their lifetime (22). As mentioned before, depression is a disease that affects women more than men (8) and is directly related to decreased BMD (20). The relationship between menopause-induced depression and osteoporosis reveals that depression is a risk factor for osteoporosis during the postmenopausal period. During this period, women with depression have lower BMD, and are more likely to have osteoporosis than non-depressed postmenopausal women (20).

## Agomelatine

Agomelatine is an antidepressant that combines non-monoaminergic signaling with the classical monoaminergic mechanism. It was first introduced to the market in 2009 and was licensed for MDD treatment in adults (23). It acts as an agonist of melatonergic-1 (MT-1) and melatonergic-2 (MT-2) receptors, and as a neutral antagonist for serotonergic (5-HT_2C_) receptors (24). Agomelatine is beneficial in MDD treatment and effective in the alleviation of sleep complaints (25). Agomelatine's monoaminergic and melatonergic mechanisms of action restore sleep and increase mood throughout the day. As a result, patients' quality of life is improved (25). Melatonergic and 5-HT_2C_ receptors are expressed in the suprachiasmatic nucleus, cerebral cortex, hippocampus, amygdala, and thalamus, which are involved in the pathophysiology of depression (26). MT-1 receptor expression has a daily rhythm regulated by daylight and the internal clock. Likewise, the expression of 5-HT_2c_ receptor mRNA has a circadian rhythm. Hence, agomelatine can be said to have a two-way effect. At night, the melatonin system dominates and favors sleep, whereas diurnal dominant 5-HT_2c_ blockade favors wakefulness (27). Interactivity between 5-HT_2c_ and MT receptors contributes to agomelatine's efficacy in depression by resynchronizing disrupted circadian rhythms (28). The binding affinities, half-life, and relative potencies of agomelatine and melatonin are presented in Table 1 (29).

**Table 1:** Some properties of melatonin and agomelatine (29).

	**Melatonin**	**Agomelatine**
Binding affinity	MT_1_: 0.085 nM	MT_1_: 0.062 nM
	MT_2_: 0.263 nM	MT_2_: 0.268 nM
Half-life	45 min	1–2 h
Protein binding	70%	95%
Relative potency	MT_1_:1	MT_1_:1
	MT_2_:1	MT_2_:1

## Agomelatine and Melatonin in Depression, Insomnia, and Bone Activity

A recent systematic review involved randomized double-blind controlled clinical trials on patients with mood disorders, including MDD. In these studies reviewed, melatonin was used as an augmentation strategy in MDD (30). Among these studies, Dolberg et al. (31) revealed that using 5–10 mg slow-release (SR) melatonin with 20 mg of fluoxetine had no effect on MDD. Similarly, Serfaty et al. (32), using 6 mg melatonin only, had no effect. A third study, however, revealed that buspirone (15 mg) combined with melatonin-SR (3 mg) elicited a more significant antidepressant effect than placebo or buspirone monotherapy (33). Consistent with these results, a systematic review and meta-analysis showed that melatonin had no therapeutic or prophylactic effect against depression or depressive symptoms (34). Therefore, melatonin may be thought to not have an antidepressant effect *per se* (35). Consequently, the antidepressant activity of melatonin has been suggested as controversial (35). According to expert opinion, the evidence supporting the clinical use of melatonin should be carefully reviewed (30). Regarding agomelatine, most reviews consensually support the idea that agomelatine has antidepressant efficacy (36–40). Moreover, according to a meta-analysis study, the antidepressant efficacy of agomelatine has been established by 20 trials with 7,460 participants that agomelatine was significantly more effective than placebo in MDD (41). Doses of 25–50 mg/day have been used in comparative experiments and placebo-controlled trials. The doses of agomelatine have been indicated to be within this range (42). However, almost no study has investigated the effects of agomelatine on MDD in menopausal women. Heun et al. (43) in comparing the effectiveness of agomelatine (25–50 mg/day p.o.) to that of placebo in an 8-week treatment schedule of elderly patients (≥ 65 years old) with recurrent MDD, proposed agomelatine (25–50 mg/day) to be more effective and well tolerated in elderly patients with depression. Additionally, agomelatine has been shown to be well tolerated by patients with only minimum deviations from placebo, and it has a good safety profile in such patients, including elderly patients (43). Besides, 69.5% of the 151 patients who received agomelatine were elderly women in this study. To the best of our knowledge, however, no study compared the antidepressant activity of agomelatine and melatonin on menopausal period in the literature. Thus, apart from melatonin, the current results suggest that agomelatine may be addressed as a reasonable choice for the elderly population, including menopausal women because of its good tolerability, effectiveness on symptoms, and better adverse effect profile.

As mentioned earlier, there is a bidirectional relationship between insomnia and MDD in menopause. Insomnia has been suggested to increase the risk of experiencing depressive symptoms by 2–3 times during the peri-menopausal period (2), and it often causes depression (11). Melatonin has been suggested to relieve all subjective sleep symptoms of postmenopausal women with insomnia (7). Additionally, melatonin has a moderate hypnotic effect in insomnia treatment and does not cause morning hangover symptoms (44–46). In the last few decades, prolonged-release melatonin (PRM) (2 mg) for insomnia with reduced sleep quality in people over 55 years old has been accepted (47, 48). It is the only melatonin-containing drug that was accepted, and its prolonged-release mechanism mimics the internal melatonin secretion pattern by progressively releasing melatonin (7). PRM (2 mg) improved sleep efficiency, morning wakefulness, and quality of life, as well as sleep latency, among people aged 55–80 years—these benefits were sustained or augmented over 6 months (47). The majority of hypnotic-related safety issues do not arise with PRM (2 mg). Accordingly, PRM (2 mg) can be suggested as a useful therapeutic option for menopausal women and stands for a safer choice over benzodiazepine or Z-drugs (7).

Regarding the effects of agomelatine on insomnia, it is suggested to have benefits with initial insomnia. Additionally, it increases sleep duration and performance, and decreases daytime drowsiness. However, it does not affect the sleep architecture of patients with depression (49). In a randomized double-blind controlled study, agomelatine presented significantly better improvement than venlafaxine on insomnia in patients with depression (28). In another randomized double-blinded study, Quera-Salva et al. (50) showed that agomelatine more significantly reduced sleep latency than escitalopram. In randomized clinical trials comparing agomelatine to selective serotonin reuptake inhibitors (SSRIs) and venlafaxine, agomelatine enhanced all parts of the sleep–wake cycle, especially during the initial stages of sleep and sleep quality, as well as daytime alertness (51). Agomelatine has also been found to be effective in alleviating circadian rhythm disturbances reported in patients with MDD (29). Melatonin treatment in menopause for insomnia has been suggested to reduce sleep disturbances during menopause. Although no study directly compares melatonin and agomelatine in the treatment of insomnia, agomelatine seems to hold promise for insomnia treatment in menopausal women.

Melatonin is known to have positive effects on bone health and is suggested to have likely therapeutic potential on postmenopausal osteoporosis (52). Li et al. (53) reviewed the role and importance of melatonin in bone metabolism. In relation to the effects of melatonin on osteoporosis, Sharan et al. (52) showed that melatonin reverses bone loss by greatly increasing bone formation in ovariectomized mice, which are the animal models of menopause-induced osteoporosis. According to the study findings, MT-2 is the receptor responsible for the effect of melatonin on osteoblasts, whereas inactivation of MT-1 does not affect bone mass. Conversely, agomelatine, as an MT-1 and MT-2 agonist, has not been studied in osteoporosis occurring among menopausal women. However, one recent study showed that agomelatine has ameliorative effects on bone fracture healing in rodents (54). Bone mass is known to be formed by either excessive osteoclastic bone resorption or reduced osteoblastic bone formation. The bone remodeling process starts by proliferation, differentiation, and activation of mononuclear precursors. Two crucial proteins have important roles in these processes: receptor activator of NF-κB ligand (RANKL) and its soluble decoy receptor osteoprotegerin (OPG). RANKL binds to its cellular receptor RANK and stimulates differentiation and activation in osteoclasts resorbing bone. Osteoprotegerin inhibits osteoclastogenesis by binding and inactivating RANKL (55). Zhou et al. (56) reported that RANKL was suppressed by melatonin. Compatible with these findings, in the same study, the OPG/RANKL ratio, which is an important determinant of bone mass and skeletal integrity (57), has been suggested to be elevated by melatonin in bone marrow mesenchymal stem cells. Additionally, NF-κB was inactivated via the MT-2 receptor, which is responsible for melatonin-regulated osteogenesis. Hence, considering all these results, agomelatine may possibly have such an effect via the MT-2 receptor on osteoporosis in menopausal women.

## The Hypothesis

Guided by literature, we hypothesize that agomelatine may be a new alternative in the combined treatment of insomnia, depression, and osteoporosis in menopausal women, treating these three disorders concurrently in comorbid conditions.

## Evaluation of the Hypothesis

Insomnia and depression are known to have a mutual relationship without clarity of the direction of effect on each other (58). This relationship also indicates that the association between depression and insomnia is not simply a cause-effect relationship, but instead a complex bidirectional one (59). Altogether, treatment of insomnia most likely may positively affect depressive symptoms in menopausal women due to their comorbidity. In a recent study supporting this situation, insomnia treatment was reported to significantly contribute to the reduction of depressive symptoms (11). Hypnotics and antidepressants are generally prescribed for the treatment of patients with comorbid of depression and sleep problems. However, hypnotics may lead to depression (59). Benzodiazepine hypnotics have many different side effects, including next-day hangover and rebound insomnia, and the next-day hangover is reported to be the most frequent complaint reported by patients using benzodiazepines. Benzodiazepines were suggested to significantly decrease sleep latency and increased total sleep duration (60). However, they are associated with significant adverse events on long-term use (60). Concerning SSRIs as a treatment for insomnia, a comprehensive review of the literature suggests that escitalopram is a safer alternative, as an SSRI, among the various antidepressants, especially with comorbid depression, for the treatment of insomnia in menopause (13).

However, SSRIs are suggested to probably increase fracture risk by decreasing BMD. Conversely, according to a US randomized placebo-controlled trial conducted that assessed the effect of escitalopram on bone turnover markers, escitalopram does not change it in short-term usage. Additionally, the trial confirmed that the result of the study could not be generalized for the long-term use of other SSRIs (61). Moreover, antidepressants in this group have many different side effects (62), and one of the most prominent of these side effects was reported to be on sleep (60). From this point of view, since agomelatine has a better side-effect profile, efficacy, and tolerability in the treatment of depression than SSRIs, as an antidepressant and sleep regulator, due to its ability to alleviate complaints about sleep disorders (60), it stands out as a new multi-target alternative for insomnia, depression, and osteoporosis treatment with minimum adverse effects.

## Conclusion

Regarding the domino effect observed in the vicious cycle involving insomnia and depression, the use of agomelatine, an antidepressant that has been proven effective in both disorders, to break this cycle would reduce both the treatment burden and global financial burden of drug consumption for patients with comorbidity insomnia and depression. Concurrently, the treatment is predicted to contribute to the improvement of osteoporosis, which seriously affects the quality of life of middle-aged women and causes bone tissue fractures in subsequent years. This bidirectional therapeutic effect provided by agomelatine is very important, since improvements in sleep in patients with depression are associated with a reduction in the recurrence rate of depressive symptoms (60). Therefore, the ameliorative effect to be achieved with agomelatine in patients with comorbid depression and insomnia would ensure that both disorders would be cured along with osteoporosis.

## Future Perspective

If our hypothesis is correct, agomelatine would come into prominence as a multi-target agent due to its curative effect on insomnia, depression, and osteoporosis, which are commonly seen in menopausal women. However, some important points need to be stressed. First, the use of agomelatine would seem to be a rational choice for middle-aged women comorbid with depression and insomnia due to its bidirectional effects. Additionally, since agomelatine has a good therapeutic potential for depression, it may have an indirect preventive role against osteoporosis, given that depression itself is an important risk factor for osteoporosis. Based on a recent study, it can as well directly heal osteoporosis through MT-2 receptors (52). Concerning agomelatine's antidepressant and sleep constructive effect in menopausal women, only one pilot study has been conducted to date (63). Seemingly, agomelatine, as a novel therapeutic avenue for being a multi-target drug, would treat all three diseases concurrently in this specific patient population, since health issues (especially for insomnia and depression) in middle-aged women are likely linked together and coexist.

## Data Availability Statement

The original contributions presented in the study are included in the article/supplementary material, further inquiries can be directed to the corresponding author/s.

## Author Contributions

AY developed the hypothesis and wrote the manuscript. MRO contributed to the literature review and manuscript editing. HK is the coordinator of the paper, he critically revised the article for important intellectual content. All authors contributed to the article and approved the submitted version.

## Conflict of Interest

The authors declare that the research was conducted in the absence of any commercial or financial relationships that could be construed as a potential conflict of interest.
